# An Oleocanthal-Enriched EVO Oil Extract Induces the ROS Production in Gastric Cancer Cells and Potentiates the Effect of Chemotherapy

**DOI:** 10.3390/antiox11091762

**Published:** 2022-09-07

**Authors:** Sara Peri, Jessica Ruzzolini, Silvia Urciuoli, Giampaolo Versienti, Alessio Biagioni, Elena Andreucci, Silvia Peppicelli, Francesca Bianchini, Andrea Bottari, Lido Calorini, Chiara Nediani, Lucia Magnelli, Laura Papucci

**Affiliations:** 1 Department of Experimental and Clinical Medicine, University of Florence, 50134 Florence, Italy; 2 Department of Experimental and Clinical Biomedical Sciences “Mario Serio”, University of Florence, 50134 Florence, Italy; 3 PHYTOLAB (Pharmaceutical, Cosmetic, Food Supplement, Technology and Analysis)-DiSIA, University of Florence, Via U. Schiff, 6, 50019 Florence, Italy; 4 Digestive Surgery Unit, AOU Careggi University Hospital, 50134 Florence, Italy; 5 Center of Excellence for Research, Transfer, and High Education (DENOTHE), University of Florence, 50134 Florence, Italy

**Keywords:** gastric cancer, chemoresistance, nutraceuticals, oleocanthal, complementary therapy

## Abstract

Oleocanthal, a minor polar compound in extra-virgin olive (EVO) oil, contains anticancer properties, which should be encouraged in its use in oncology. Gastric Cancer (GC), a very aggressive human cancer, is often diagnosed at advanced stages, when surgery is substituted or supported by chemotherapy (CT). However, CT frequently fails due to the patient’s resistance to the treatment. Thus, the aim of this study is to verify whether an OC-enriched EVO oil extract fraction (OCF) may be useful in order to overcome a resistance to GC. We evaluated the OCF effects on an AGS gastric adenocarcinoma cell line wild type (AGS wt) and on its subpopulations resistant to 5-fluorouracil (5FUr), Paclitaxel (TAXr) or cisplatin (CISr). We found that a 60 µM dose of the OCF acts on the AGS wt, 5FUr and TAXr, leading to the cell cycle inhibition and to a ROS production, but not on CISr cells. Resistance of CISr to the OCF seems to be due to higher levels of antioxidant-enzymes that can counteract the OCF-induced ROS production. Moreover, using the OCF plus 5-fluorouracil, Paclitaxel or cisplatin, we found a potentiating effect compared with a mono-treatment in all resistant GC cells, including CISr. In conclusion, the use of the OCF in the management of GC has shown very interesting advantages, opening-up the possibility to evaluate the efficacy of the OCF in vivo, as a valid adjuvant in the treatment of resistant GC.

## 1. Introduction

Cancer is emerging as a prominent public health issue representing the second leading cause of death worldwide [1]. Despite numerous advances in medical sciences, off-target toxicity and drug resistance remain potential challenges in clinical oncology. Considerable attempts have been made in order to improve the conventional therapeutic approaches with the use of radiotherapy, immunotherapy, chemotherapy and surgical methods [2]. Unfortunately, chemotherapy has collateral adverse effects, such as cardio- and hepatotoxicity, nephron- and neurotoxicity and life-threatening hematopoietic toxicity that restrict its use [2].

In this context, naturally occurring compounds from plants, known as phytochemicals, are emerging as potential novel anticancer drugs. Approximately 50% of approved anticancer drugs from 1940 to 2014 originated from natural products or directly derived from these [3]. Phytochemicals including polyphenols, exhibit anticancer properties due to their anti-metastatic, anti-proliferative, anti-angiogenic, anti-inflammatory, cell cycle arrest, apoptotic and autophagic effects [4]. Polyphenols can act as chemopreventive compounds, as they often have a similar structure and/or identical mechanisms with molecularly targeted chemotherapeutic agents. Therefore, polyphenols may be used as alternatives or complementary coadjuvants to conventional antitumoral therapies, thereby potentiating anticancer activities and diminishing chemotherapy-associated toxicity at the same time [5,6].

Extra-virgin olive (EVO) oil, one of the most characteristic components of a Mediterranean diet [7,8,9], is known for its nutritional properties and health effects and its intake is associated with the reduced risk of many diseases related to aging, including some cancers [4], in particular those of the gastrointestinal tract. The beneficial properties of EVO were attributed to the presence of high levels of fatty acids, in particular monounsaturated acids such as oleic acid, as well as of other bioactive components known as minor polar compounds (MPCs) including polyphenols [10]. An important MPC of EVO oils, discovered in 1992 by Montedoro et al. [11,12], is Oleocanthal (OC) [(-)-deacetoxyligstroside aglycone], which is responsible for the sensation of irritation and pungency in the throat and induced by EVO oil. Although structurally dissimilar [13], OC shows an anti-prostaglandin-related anti-inflammatory effect similar to that of the non-steroidal anti-inflammatory drug (NSAID), Ibuprofen [14], and for this reason OC is considered a naturally occurring NSAID [15,16,17,18].

In addition to its anti-inflammatory activity, OC reveals several biological properties that encourage its use in various medical fields, such as oncology.

*Toric* et al. [19] collected much evidence regarding the anticancer effects of olive oil polyphenols and OC, in particular, has been found to be active in several types of cancer, such as breast cancer [18,20,21,22], melanoma [23,24], prostate cancer [18], hepatocellular cancer [25], colorectal cancer [26], multiple myeloma [27] and non-melanoma skin cancers [28]; however, currently, no data has been reported on the effects of OC on gastric cancer (GC), a very aggressive and therapy-resistant type of human cancer.

Despite some progress over the past half century, GC remains a highly incident tumor, representing the fifth most common malignant lesion worldwide and the fourth cause of tumor-related deaths [1]. In most cases (>70% cases), the lack of specific signs and symptoms allows for the diagnosis of GC only once the disease is in an advanced stage, thereby restricting the therapeutic strategies. However, the 5-year survival rate drastically increases in countries where, due to the high incidence of GC, they carry out mass screening campaigns, such as in Japan and Korea (>90%) [29,30], while it remains low in the others (10–42.9%) [31,32] with a median overall survival rate for metastatic GC of less than 1 year [33]. Surgery remains the gold standard for the treatment of GC and alternative chemotherapy or radiotherapy can be used in advanced stages, in either a pre-, peri- or post-operative setting [34]. Chemotherapy regimens are usually based on the combination of unspecific drugs such as fluorinated-pyrimidines, platinum- or taxane-based agents (the so-called FLOT regimen) [35], which, however, do not exclude the development of chemoresistance that can cause a relapse and progression of the disease.

The aim of this study is to verify whether an OC-enriched EVO oil extract fraction (OCF) could be useful in order to affect the viability of GC cells and overcome a GC resistance, revealing the possibility of the use of the OCF in a complementary therapy of GC.

## 2. Materials and Methods

### 2.1. Extract from EVO Oils

In order to obtain an extract fraction of *Olea europaea* L. enriched in OC, OCF, Tuscan EVO oils have been used. In particular, we selected the EVO oils of the Leccino, Frantoio and Moraiolo cultivars, all of which were obtained from two-phase mills. The blend of the EVO oils used for the extraction has a total MPC content of 856 mg/kg (HPLC-DAD-MS) (Table 1). The extract tested in this work was obtained with the liquid/liquid multiple extraction method, starting from 10 L of EVO oil. The selected EVO oil blend was extracted with a 70%-EtOH:H_2_O solution at pH: 3.2 for formic acid, in the dark for 30 min at RT in a mechanical orbital shaker. The solution was transferred to a separatory funnel in order to remove the lipid portion with hexane. The liquid extract was dried under vacuum (Laborota 4000, Heidolph, Schwabach, Germany) and taken up in 50 mL of a 70%-EtOH:H_2_O solution at pH: 3.2 for formic acid. The obtained extracts were mixed and dried again and then taken up in 50 mL of H_2_O. The final redissolved extract was then degreased twice with hexane, directly in the test tube and placed in an HPLC-DAD-MS analysis for the qualitative and quantitative characterization reported below (Table 1).

### 2.2. HPLC-DAD-MS Analysis

The qualitative and quantitative evaluations of the bioactive compounds of the OCF from the EVO oils was performed using an HP-1260 liquid chromatograph equipped with a DAD detector (Agilent-Technologies, Palo Alto, CA, USA). The HPLC system is interfaced with an Agilent MS system equipped with an ESI source (Agilent Corp, Santa Clara, CA, USA). The analyses were carried out in full-scan mode and the mass range was set to *m*/*z* 100–1500 in negative modes. The analytical columns and chromatographic methods are described in *Romani* et al. [36]. The polyphenols found in the extracts were identified by comparing their retention times and their UV/Vis spectra with those of the authentic standards. Each compound was quantified at the selected wavelength (240, 280, 330, 350 nm) using a five-point regression curve and applying the correction of the molecular weights [36].

### 2.3. Cell Culture

The human microvascular endothelial cells (HMVECs) were purchased from Lonza and were cultured in an EGM-2 culture medium (Lonza), supplemented with 10% FBS. The neonatal human dermal fibroblasts (NHDFs) obtained from Lonza were grown in a high-glucose (4500 g/L) Dulbecco’s Modified Eagle Medium (DMEM), supplemented with 10% FBS, 100 units/mL penicillin, 100 µg/mL streptomycin and 2 mM L-glutamine, (Euroclone, Milan, Italy).

The gastric adenocarcinoma cell line AGS (AGS wt) was purchased from ATCC (CRL-1739) and it was originally obtained from the primary tumor of a 54 year old female patient who had not received any chemotherapy treatment prior to explantation. The AGS cells that are chemoresistant to 5-fluorouracil (5FUr), Paclitaxel (TAXr) and cisplatin (CISr) were obtained, as previously described by *Peri* et al. [37].

The AGS wt, 5FUr, TAXr and CISr cell lines were cultured in F-12K Nutrient Mixture Kaighn’s Modification (Corning, distributed by Sial, Rome, Italy) supplemented with 10% heat inactivated FBS (Euroclone, Milan, Italy) and 100 U/mL penicillin and 100 µg/mL streptomycin (Euroclone, Milan, Italy), in a humidified atmosphere with 5% CO_2_ at 37 °C. The cells were passed before reaching confluence by trypsinization, then washed in PBS and plated in new vessels with a completely fresh medium. The resistant cells were cultured every other week in the presence of 40 µM 5-fluorouracil (Sigma Aldrich, Milan, Italy) for 5FUr, 10 nM Paclitaxel (Sigma Aldrich, Milan, Italy) for TAXr and10 µM cis-Diammineplatinum(II) dichloride (Sigma Aldrich, Milan, Italy) for CISr.

### 2.4. MTT Assay

The 5 × 10^3^ cells/well were seeded in a 96-well plate in a completely fresh medium. One day later, the cells were treated with the desired concentration of drugs and after 72 h, the medium was removed and the cells were incubated for 1.5 h with completely fresh media without phenol red supplemented with 0.5 mg/mL MTT (3-(4,5-dimethyl-2-thiazolyl)-2,5-diphenyl-2H-tetrazolium bromide; M5655 Sigma Aldrich). The cells were then lysed in 100 µL of DMSO (Sigma Aldrich, Milan, Italy). The blue formazan absorbance was automatically read at 570 nm using a spectrophotometric microplate reader (Biorad, Milan, Italy). The results were analyzed using the Graphpad Prism software.

### 2.5. Cytofluorimetric Annexin V/PI Double Staining

The 8 × 10^7^ cells were plated in p60 dishes and the following day were treated with drugs. Following 72 h of treatment, the media were collected in 5 mL flow cytometry tubes and the cells were detached with Accutase (Euroclone, Milan, Italy). The cells were then washed with an annexin binding buffer (10 mM Hepes pH 7.4; 140 mM NaCl; 8 mM CaCl_2_) and stained with 3 µL of annexin V-APC (Immunotools, Friesoythe, Germany) and 1 µL of propidium iodide 0.1 mg/mL (Sigma Aldrich, Milan, Italy) in 100 µL of an annexin binding buffer for 30 min at RT. The cells were analyzed with BD FACSCanto II and analyzed using FlowJo software (BD Biosciences, distributed by DBA, Milan, Italy).

### 2.6. Plate Colony Forming Assay

Following 72 h of different OCF concentration treatments, the cells were counted using the trypan blue exclusion test and an equal amount of cell volume established on control (EtOH) was transferred in a fresh medium and incubated for 10 days at 37 °C. The colonies were washed with PBS, fixed in cold methanol and stained using a Diff Quik kit (BD Biosciences, distributed by DBA, Milan, Italy). The stained colonies were photographed with a digital camera and the number of colonies in each well was counted.

### 2.7. Western Blotting

The cells were lysed after the 24 h treatment and the proteins were separated using electrophoresis, as previously described [38]. The primary antibodies were: rabbit anti-p21 (1:1000, Cell Signaling Technology, distributed by Euroclone, Milan, Italy), rabbit anti-phospho Rb (1:1000, Cell Signaling Technology) and rabbit anti Rb (1:1000, Cell Signaling Technology). The membrane was washed in a T-PBS buffer, then incubated for 1 h at RT with a goat anti-rabbit IgG Alexa Fluor 750 antibody or with a goat anti-mouse IgG Alexa Fluor 680 antibody (Invitrogen, Milan, Italy) and then visualized using an Odyssey Infrared Imaging System (LI-COR^®^ Bioscience, distributed by Carlo Erba, Milan, Italy). The rabbit anti-vinculin (1:1000, Cell Signaling Technology) was used in order to assess the equal amounts of protein loaded in each lane.

### 2.8. ROS Assessment Using a Flow Cytometry Analysis

The 8 × 10^7^ cells were seeded in p60 petri dishes and one day later, they were treated with 60 µM of OCF. Following a period of 72 h, the cells were detached using trypsin-EDTA (Euroclone, Milan, Italy), centrifuged at 400× *g* for 5 min and then re-suspended in 300 µL of a complete medium that was pre-equilibrated at 37 °C with 10 µM of DCFDA (Sigma Aldrich, Milan, Italy). The cells were then incubated for 30 min at 37 °C, in the dark. A positive control sample was prepared adding 20 mM H_2_O_2_ during the incubation with the dye. The cells were then promptly analyzed through a BD FACSCanto II Flow Cytometer detecting DCFDA fluorescence in the FITC channel. Using the FlowJo software, a gating for DCFDA positivity was set in non-treated cells in order to have 95% negative and 5% DCFDA-positive cells. The same gate was used for the OCF-treated cells and the increment in % of positive cells is reported in Figure 10a.

### 2.9. Cytofluorimetric Intracellular Staining

The cells, treated for 72 h with the OCF as reported above, were detached with Accutase (Euroclone, Milan, Italy), then washed twice in PBS and resuspended in 4% PFA in PBS. Following an incubation at 4 °C for 10 min, the cells were centrifuged at 400× *g* for 5 min and the pellet was resuspended in PBS 0.25% Triton™ X-100 (Sigma Aldrich, Milan, Italy) for 5 min. The cells were pelleted again and incubated O/N at 4 °C with a 2 µg/µL anti-p53 mouse primary antibody (1:100, Santa Cruz Biotechnology, distributed by DBA, Milan, Italy) in PBS 0.1% Triton™ X-100 0.2% BSA. The cells were then washed in PBS twice and incubated for 1 h at RT with an anti-mouse antibody conjugated with FITC (1:200, Merck Millipore, Milan, Italy) in PBS 0.1% Triton™ X-100 0.2% BSA. The cells were washed twice in PBS, resuspended in PBS and analyzed with a BD FACSCanto II flow cytometer.

### 2.10. qRT-PCR

The AGS wt and chemoresistant cell lines were cultured in p100 petri dishes with a complete medium and before they reached confluence, they were lysed in 500 µL of a TRI Reagent (Sigma Aldrich). Following the manufacturers’ instructions, the RNA was extracted, solubilized in RNase/DNase free water and dosed with a NanoDrop™ One/OneC Microvolume UV-Vis Spectrophotometer (Thermofisher, Monza, Italy). 1 µg of RNA was then retrotranscribed to the cDNA using the iScript™ cDNA Synthesis Kit (Biorad, Milan, Italy) and following the manufacturers’ instructions. The cDNA was then diluted 1:5 in RNase/DNase free water. The real time PCR was performed in a CFX96 Touch Real-Time PCR Detection System (Biorad, Milan, Italy) using the primers listed below (Table 2). 1 µL 4 µM of forward and reverse primers mix, 2 µL of diluted cDNA, 2 µL of water and 5 µL of SsoAdvanced Universal SYBR Green Supermix were dispensed into a 96 well plate with each experimental point in triplicate. Real time was performed with the following steps: (1) 2 min at 95 °C, (2) 15 s at 95 °C, (3) 30 s at 60 °C, (4) repeat from step 2, 39 more times (5) and then increase the temperature from 55 °C to 95 °C, increasing 0.5 °C/s. The data were then analyzed using the CFX Maestro software (Biorad, Milan, Italy).

### 2.11. Statistics

The results are obtained from at least three independent experiments and expressed as means ± SD. The GraphPad Prism program was used in order to perform multiple comparison tests as specified in each figure legend. The statistical significance was accepted at *p* < 0.05.

## 3. Results

The aim of this study is to test whether an OC-enriched fraction of EVO oil (OCF), is effective on AGS gastric cancer cells (wt) and on AGS drug-resistant cells were previously selected in our laboratory by exposing the AGS cells to 5-fluorouracil, Paclitaxel or cisplatin [37], which are the classic components of GC polychemotherapy.

### 3.1. OCF Effects on Normal Cells and on GC AGS Wild Type and Resistant Cells

**Figure 1:**
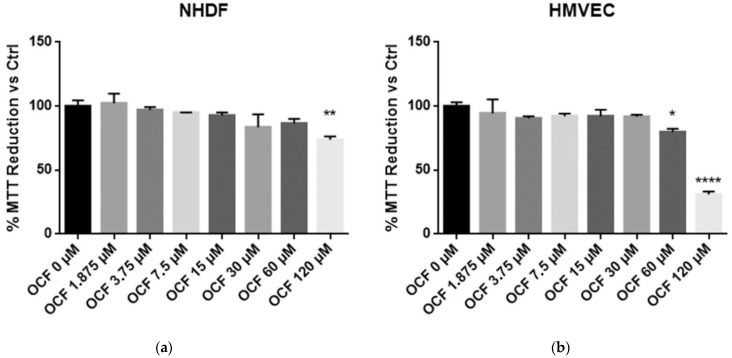
MTT assay on normal cells. NHDF (Neonatal Human Dermal Fibroblasts) (**a**) and HMVEC (Human Microvascular Endothelial Cells) (**b**) are non-transformed cell cultures and these were used in order to test their vitality using an OCF dose-dependent treatment through a MTT test. Data are presented as mean ± SD; * *p* < 0.05; ** *p* < 0.01; **** *p* < 0.0001 (One-way ANOVA Dunnett’s multiple comparison test).

**Figure 2:**
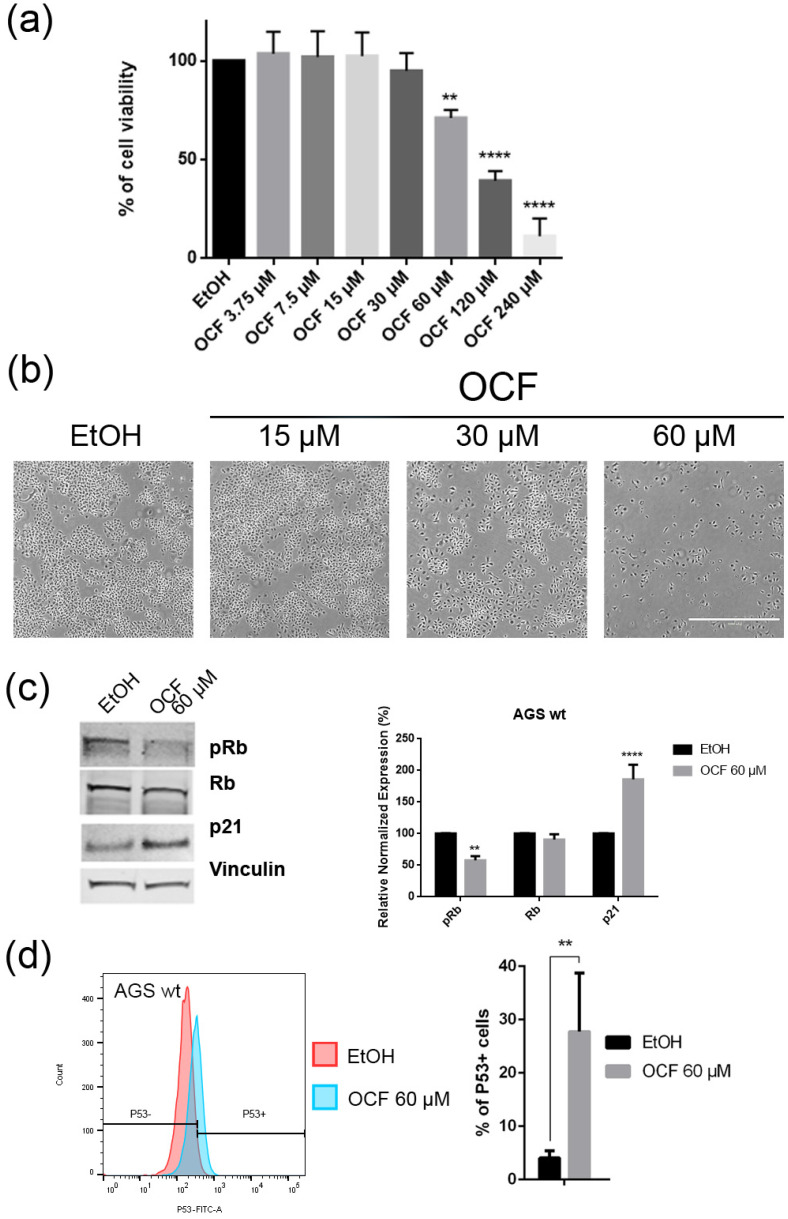
Effects of different doses of OCF on AGS wt cells. AGS wt cells, treated for 72 h with vehicle or different doses of the OCF, were analyzed through a MTT assay (**a**); pictures were obtained with an inverted microscope at 4× magnification (**b**); representative western blot analysis of phosphor-Rb (p-Rb), Rb, p21 and vinculin levels in the AGS wt treated with vehicle or 60 µM dose of the OCF for 24 h and the corresponding densitometry (**c**). Flow cytometry plots for the p53 staining and the corresponding histograms (**d**). Results of the representative experiments are shown. ** *p* < 0.01, **** *p* < 0.0001. (**a**) One-way ANOVA Dunnett’s multiple comparisons test; (**c**,**d**) Two-way ANOVA Sidak’s multiple comparisons test.

**Figure 3:**
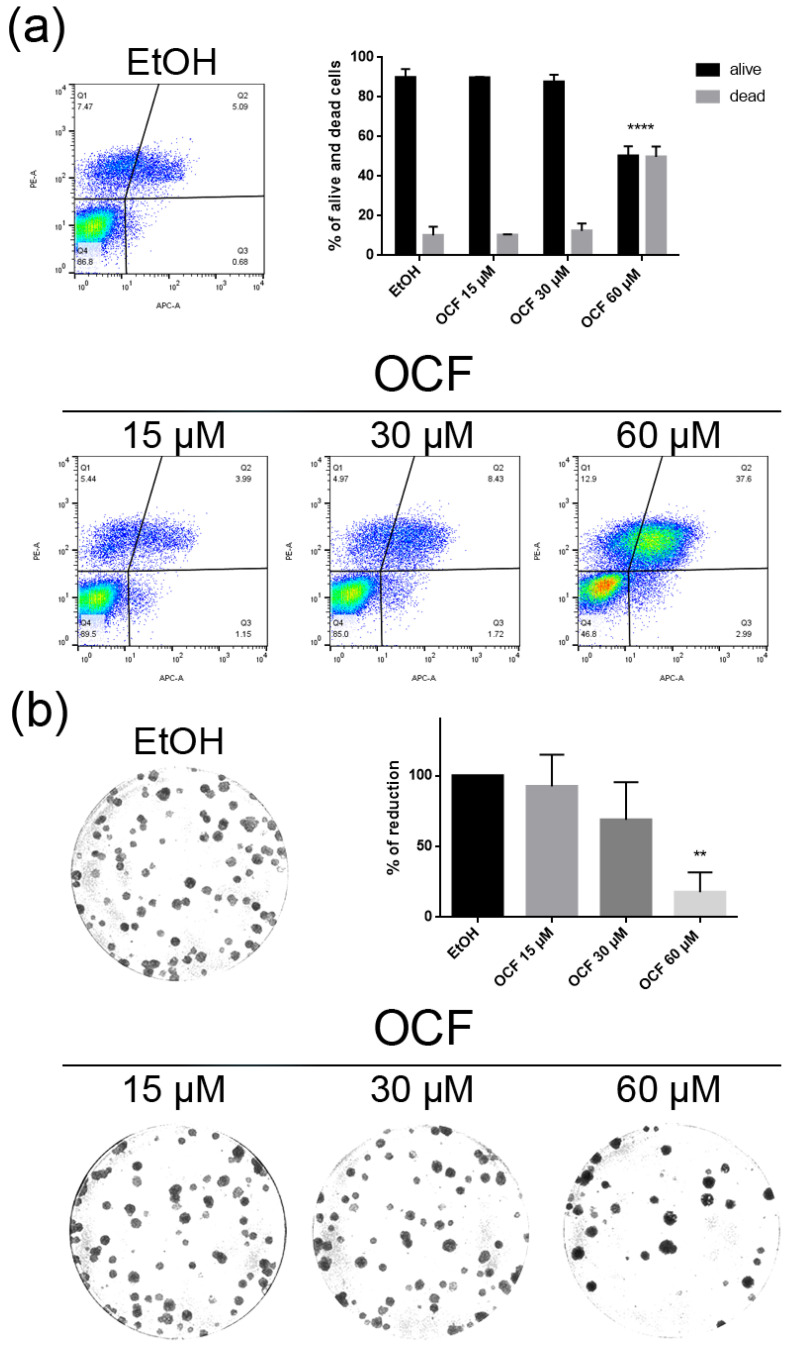
Vitality and colony formation assay of the OCF-treated AGS wt cells. AGS wt cells were treated for 72 h with vehicle or different doses of the OCF; plots and corresponding histograms for the live/dead cells percentages obtained using annexin V/PI double staining, are shown (**a**). Cell colonies obtained after 72 h of the vehicle/OCF treatment and corresponding percentage quantification (**b**). Data are presented as mean ± SD of three independent experiments. Results of the representative experiments are shown. ** *p* < 0.01, **** *p* < 0.0001. (**a**) Two-way ANOVA Tukey’s multiple comparisons test; (**b**) One-way ANOVA Dunnett’s multiple comparisons test.

**Figure 4:**
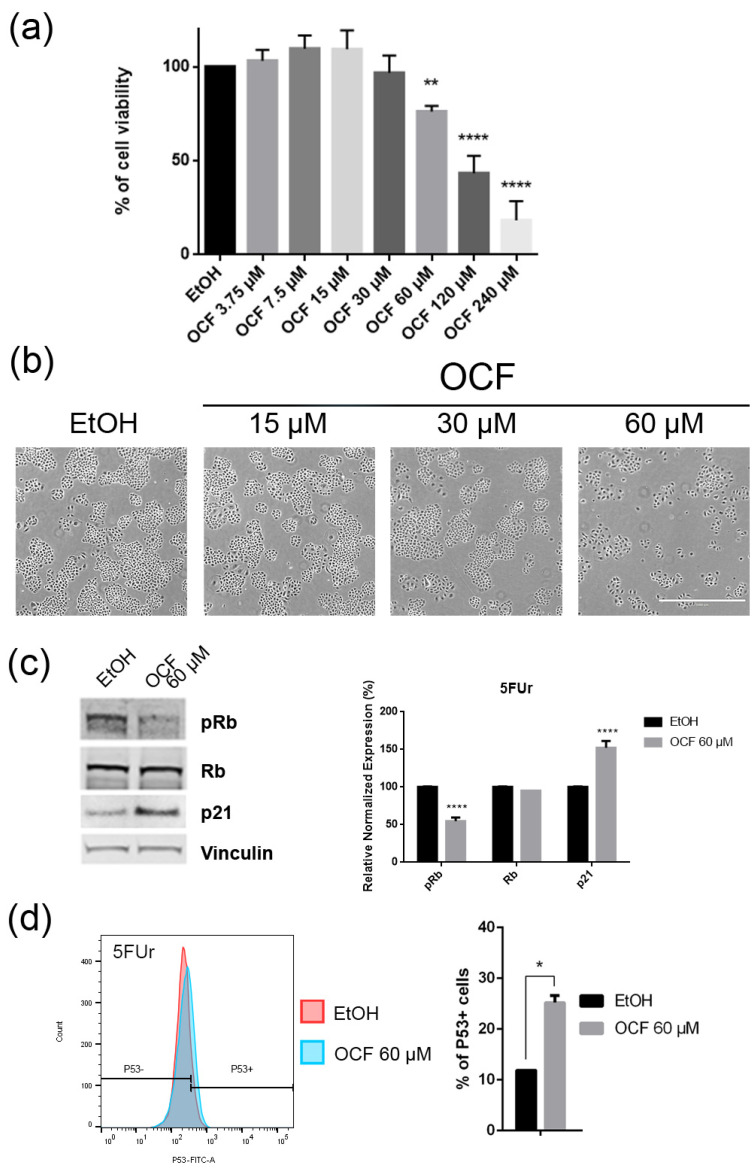
Effects of different doses of OCF on the 5FUr cells. 5FUr cells, treated for 72 h with vehicle or with different doses of the OCF, were analyzed through a MTT assay (**a**); pictures were obtained with an inverted microscope at 4x magnification (**b**); representative western blot analysis of p-Rb, Rb, p21 and vinculin levels in 5FUr cells were treated with vehicle or 60 µM dose of the OCF for 24 h and the corresponding densitometry (**c**). Flow cytometry plots for the p53 staining and corresponding histograms (**d**). Data are presented as mean ± SD of three independent experiments. Results of the representative experiments are shown. * *p* < 0.05, ** *p* < 0.01, **** *p* < 0.0001. (**a**) One-way ANOVA Dunnett’s multiple comparisons test; (**c**,**d**) Two-way ANOVA Sidak’s multiple comparisons test.

**Figure 5:**
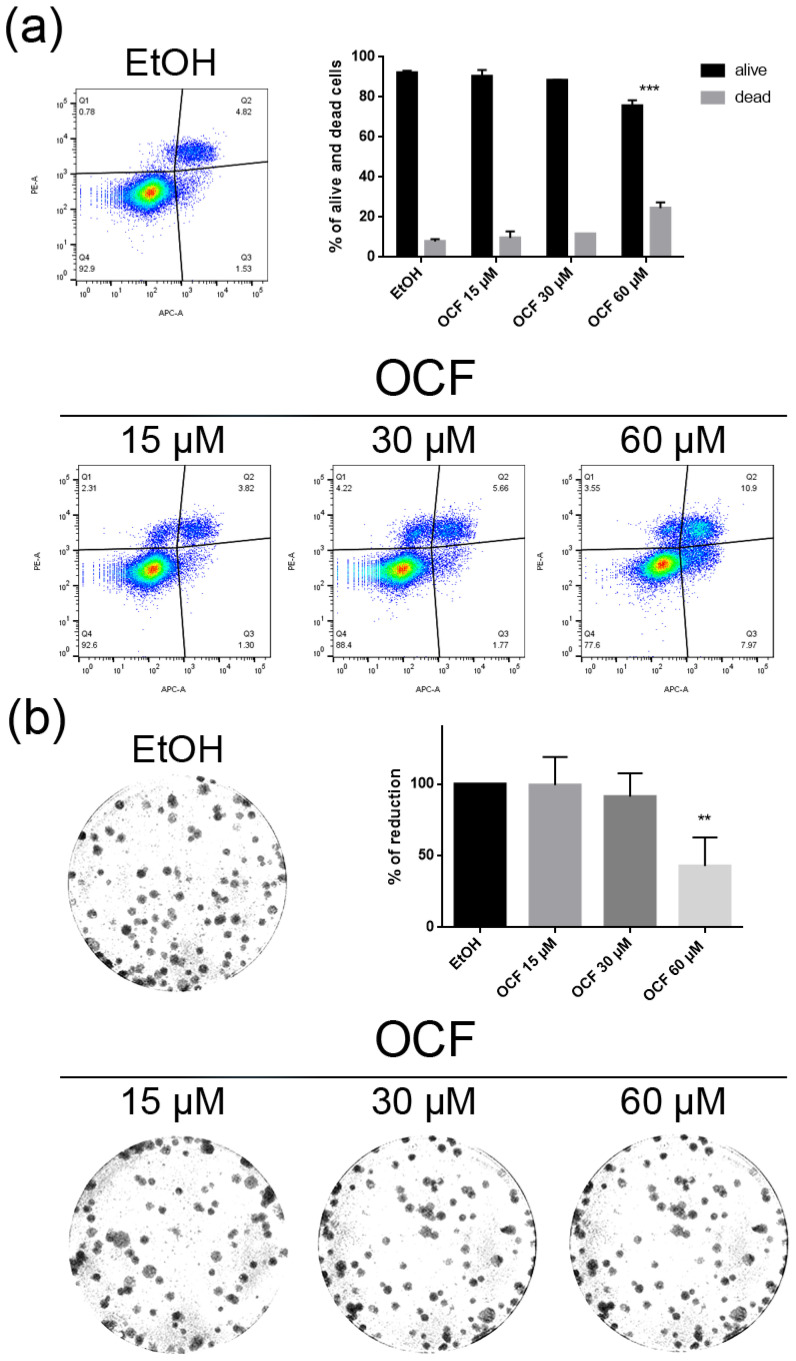
Vitality and colony formation assay of the OCF-treated 5FUr cells. 5FUr cells were treated for 72 h with vehicle or with different doses of the OCF; plots and corresponding histograms for live/dead cells percentages obtained by an annexin V/PI double staining are shown (**a**). Cell colonies obtained after 72 h of the vehicle/OCF treatment and the corresponding percentage quantification (**b**). Data are presented as mean ± SD of three independent experiments. Results of the representative experiments are shown. ** *p* < 0.01, *** *p* < 0.001. (**a**) Two-way ANOVA Tukey’s multiple comparisons test; (**b**) One-way ANOVA Dunnett’s multiple comparisons test.

**Figure 6:**
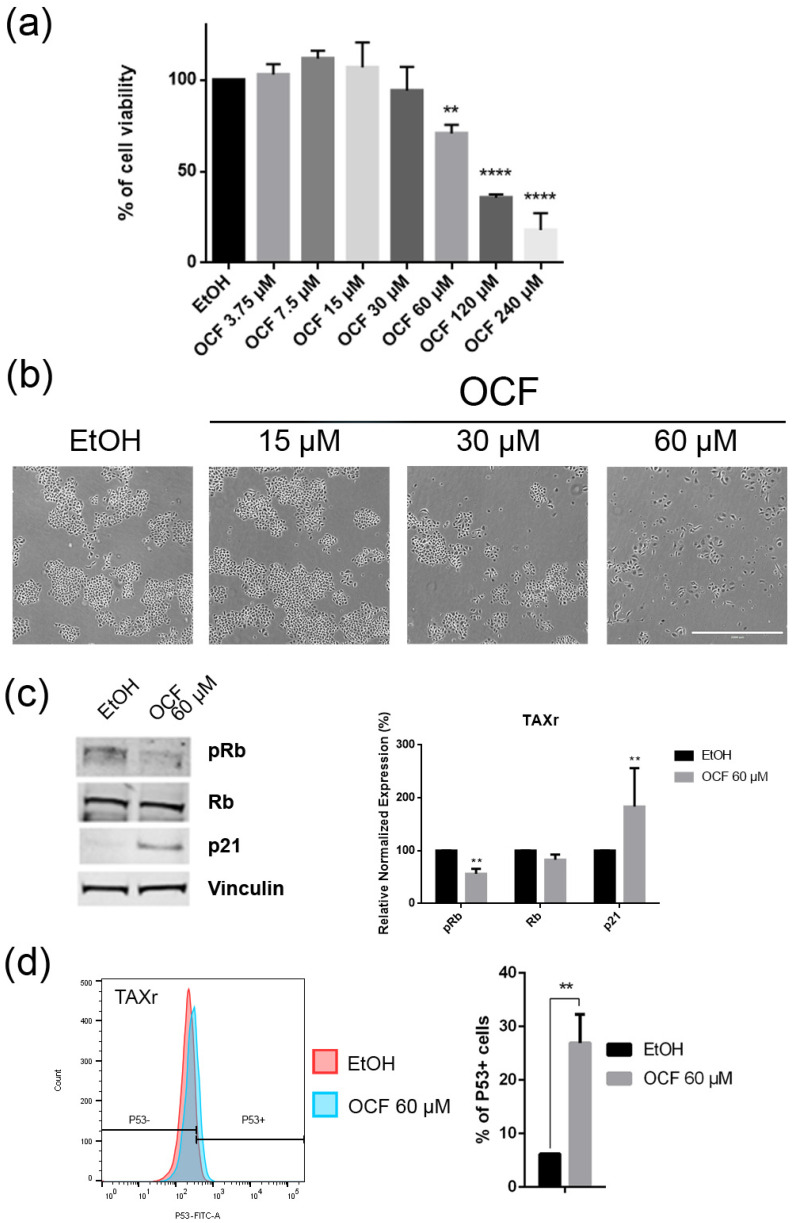
Effects of the different doses of the OCF on TAXr cells. TAXr cells, treated for 72 h with vehicle or with different doses of the OCF, were analyzed through a MTT assay (**a**); pictures were obtained with an inverted microscope at 4x magnification (**b**); representative western blot analysis of p-Rb, Rb, p21 and vinculin levels in the TAXr cells treated with vehicle or 60 µM dose of the OCF for 24 h and the corresponding densitometry (**c**). Flow cytometry plots for the p53 staining and the corresponding histograms (**d**). Data are presented as mean ± SD of three independent experiments. Results of the representative experiments are shown. ** *p* < 0.01, **** *p* < 0.0001. (**a**) One-way ANOVA Dunnett’s multiple comparisons test; (**c**,**d**) Two-way ANOVA Sidak’s multiple comparisons test.

**Figure 7:**
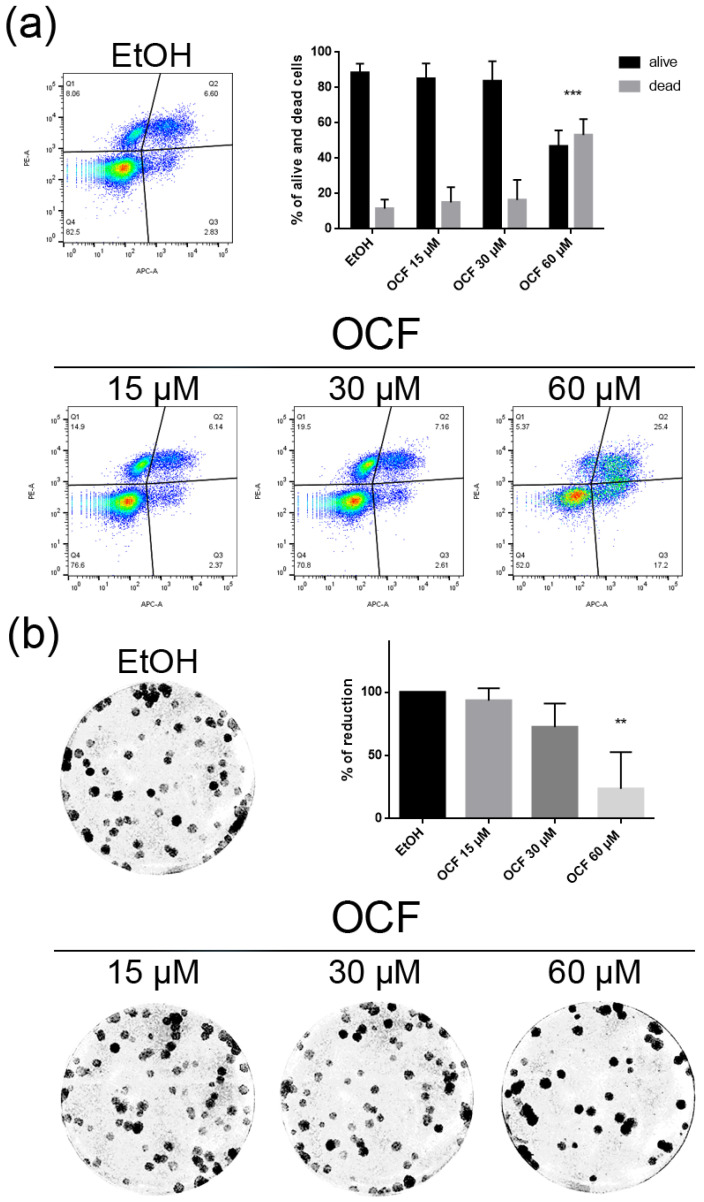
Vitality and the colony formation assay of the OCF-treated TAXr cells. TAXr cells were treated for 72 h with vehicle or with different doses of the OCF; plots and corresponding histograms for live/dead cells percentages obtained using an annexin V/PI double staining are shown (**a**). Cell colonies obtained after 72 h of the vehicle/OCF treatment and the corresponding percentage quantification (**b**). Data are presented as mean ± SD of three independent experiments. Results of the representative experiments are shown. ** *p* < 0.01, *** *p* < 0.001. (**a**) Two-way ANOVA Tukey’s multiple comparisons test; (**b**) One-way ANOVA Dunnett’s multiple comparisons test.

**Figure 8:**
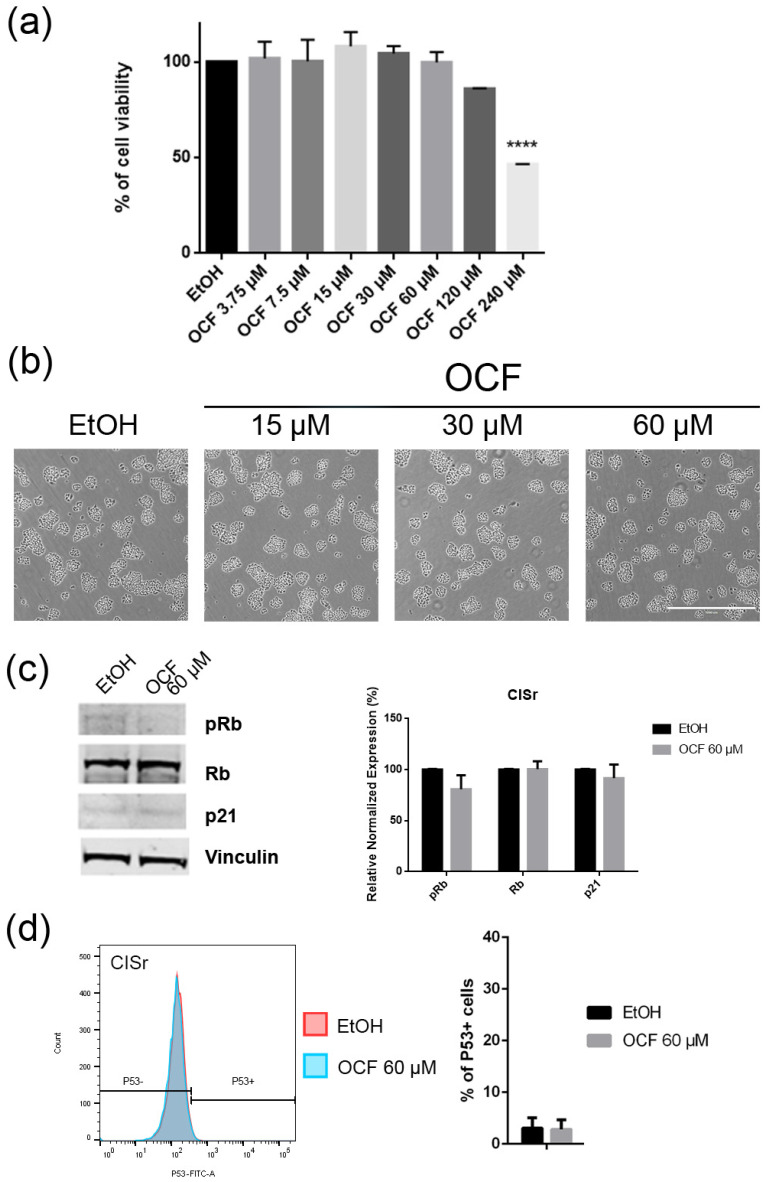
Effects of different doses of OCF on the CISr cells. CISr cells treated for 72 h with vehicle or with different doses of the OCF, were analyzed through a MTT assay (**a**); pictures were obtained with an inverted microscope at 4× magnification (**b**); representative western blot analysis of p-Rb, Rb, p21 and vinculin levels in the CISr cells treated with vehicle or with 60 µM dose of the OCF for 24 h and the corresponding densitometry (**c**). Flow cytometry plots for the p53 staining and the corresponding histograms (**d**). Data are presented as mean ± SD of three independent experiments. Results of the representative experiments are shown. (**a**) One-way ANOVA Dunnett’s multiple comparisons test; (**c**,**d**) Two-way ANOVA Sidak’s multiple comparisons test. **** *p* < 0.0001.

**Figure 9:**
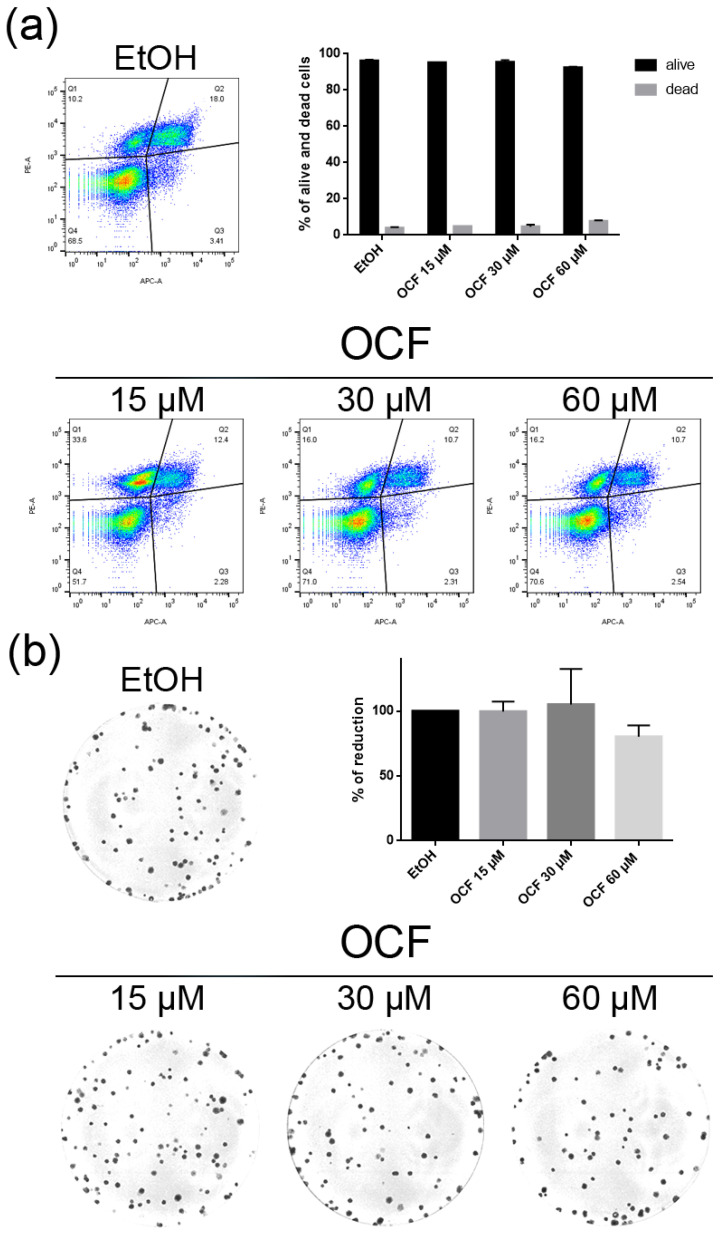
Vitality and the colony formation assay of the OCF-treated CISr cells. CISr cells were treated for 72 h with vehicle or with different doses of the OCF; plots and corresponding histograms for live/dead cells percentages obtained by an annexin V/PI double staining are shown (**a**). Cell colonies obtained after 72 h of the vehicle/OCF treatment and the corresponding percentage quantification (**b**). Data are presented as mean ± SD of three independent experiments. Results of the representative experiments are shown. (**a**) Two-way ANOVA Tukey’s multiple comparisons test; (**b**) One-way ANOVA Dunnett’s multiple comparisons test.

In order to evaluate an OCF potential toxicity, the vitality of normal cells, NHDF and HMVEC, was first tested showing a mild to moderate toxicity starting from 120 µM of OCF for the NHDF and 60 µM of OCF for the HMVEC (Figure 1). The high sensitivity of the endothelial cells to olive-leaf extract polyphenols, in respect to other normal cell lines, was previously reported by *Goulas* and coll. [39]. Therefore, the OCF concentration we selected in order to treat the AGS wt cells (Figure 2 and Figure 3), the 5-fluorouracil- (Figure 4 and Figure 5), Paclitaxel- (Figure 6 and Figure 7) and cisplatin-resistant (Figure 8 and Figure 9) cells, was 60 µM.

When the AGS wt cells were treated with the OCF, a significant dose dependent decrease of the viability starting from 60 μM (Figure 2a) was observed, as assessed by the MTT assay. This was also confirmed by a visible reduction in cell numbers (Figure 2b), which is probably due to the cell growth inhibition, as the decrease in the p-Rb levels and the increase in the p21 expression suggest (Figure 2c). In order to better understand the effects of a 60 µM dose of the OCF on the AGS wt cells, we evaluated the expression of p53, a transcription factor activated by cell stress that plays a key role in cell destiny modulating cell cycles. A significant upregulation in the p53 protein level was found (Figure 2d), suggesting that the OCF may induce apoptosis of the AGS wt cells. Indeed, the cytofluorimetric annexin V/PI assay (Figure 3a) showed a significant increase in the cellular apoptosis of the OCF-treated AGS wt cells. The cellular ability to form colonies after any type of treatment is a crucial step in tumor relapse, thus, the discovery of agents capable of preventing it would be very important. The clonogenic capacity of the AGS wt treated with a 60 µM dose of the OCF was then analyzed and a significant reduction in colony formation was found (Figure 3b). All together, these data highlight the ability of the OCF to affect the cell viability and the cloning efficiency of AGS wt cells.

The OCF was also administered to 5FUr, TAXr and CISr cells. Our model system of 5FUr cells is 1000 times more resistant to 5-fluorouracil than parental cells, as previously reported by *Peri* et al. [37]. The OCF-treated 5FUr cells showed a reduced viability starting from a 60 μM dose of the OCF (Figure 4a). At the same dose, a decrease in cell numbers is also evident (Figure 4b). The decrease in proliferation is confirmed by the analysis of the molecular markers involved in growth, such as the p21 protein expression increase and the Rb phosphorylation decrease (Figure 4c), as well as the increase in the p53 expression (Figure 4d). The annexin V/PI assay corroborates the efficacy of the 60 μM dose of the OCF in inducing cellular apoptosis (Figure 5a), although the effect was less evident than in the AGS wt cells. The clonogenic assay revealed a significant reduction of the colony numbers in the OCF-treated 5FUr (Figure 5b).

The TAXr cells were about 100 times more resistant to Paclitaxel than the AGS wt, as previously reported [37]. When the TAXr cells were treated with the OCF, we found a significant decrease in the cell viability starting from the 60 μM dose of the OCF (Figure 6a), which was associated with a reduction in the cell proliferation, an increase in the p21 (Figure 6c) and p53 protein levels (Figure 6d) and a p-Rb decrease (Figure 6c), as already reported for the AGS wt and 5FUr cells. The OCF also affected the TAXr cell apoptosis (Figure 7a) and the colony-formation ability (Figure 7b) in a similar manner as to that observed for the AGS wt and 5Fur cells.

The CISr cells were about 5 times more resistant to cisplatin than the AGS wt parental cells [37]. However, by exposing the CISr to the 60 μM dose of the OCF, no change in viability was found. Only the 240 μM dose of the OCF showed an effective 50% toxicity (Figure 8a), which however, represented a toxic dose even for normal cells (Figure 1). Moreover, in the CISr cells exposed to the 60 μM dose of the OCF, no significant changes were detected in the cell cycle markers (Figure 8c,d), the apoptosis (Figure 9a) and the colony formation assay (Figure 9b).

On the whole, these results suggest that the 60 μM dose of the OCF was effective on the AGS wt, 5FUr and TAXr cells, but not on the CISr cells.

### 3.2. Pro-Oxidant Activity of the OCF on GC Cells

**Figure 10:**
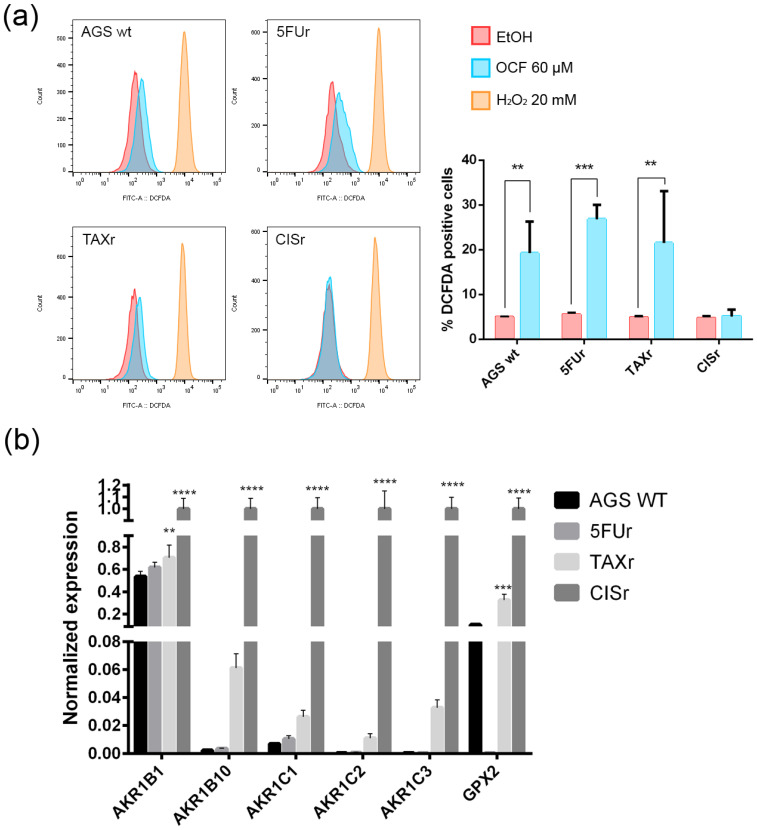
ROS production by the OCF treatment and the antioxidant enzymes levels. Flow cytometry graphs and the corresponding ROS quantification in the cells treated for 72 h with vehicle or with a 60 µM dose of the OCF (**a**). Real-Time PCR analyses for the antioxidant enzymes in the AGS wt, 5FUr, TAXr and CISr cells (**b**). Data are presented as mean ± SD of three independent experiments. Results of the representative experiments are shown. ** *p* < 0.01, *** *p* < 0.001; **** *p* < 0.0001; (**a**) two-way ANOVA Sidak’s multiple comparisons test; (**b**) Two-way ANOVA Dunnett’s multiple comparisons test.

The following literature indications reported an increase in the production of reactive oxygen species (ROS) in OC-treated colorectal and hepatocellular carcinoma cells, see *Cusimano* et al. [26]. We decided to verify whether the OCF-dependent toxicity observed in our cells may depend on the ROS production. The OCF-dependent ROS production may also be suggested by the increased level of p53 following the OCF treatment (Figure 2d, Figure 4d and Figure 6d); indeed, p53 is implicated in the cellular response of several sources of stress, including the ROS [40]. We verified that the 60 µM dose of the OCF induced the ROS increase in all of the tested cells except for the CISr cells, as revealed by the flow cytometry DCFDA assay (Figure 10a). Considering that the AGS wt, 5FUr and TAXr cells showed lower expression levels of a number of antioxidant genes, such as AKR1B, AKR1B10, AKR1C1, AKR1C2, AKR1C3 and GPX2, while the CISr expressed a high level of these protective genes, it is possible that the lack of the OCF-induced ROS production in the CISr cells may depend on this specific feature (Figure 10b).

Overall, these data suggest a role of the ROS in the OCF toxicity in our GC cells, with the exception of the CISr cells, as characterized by the high levels of antioxidant genes, which are drivers in counteracting the ROS.

### 3.3. Effects of the Combined Treatment of the OCF Plus 5-Fluorouracil, Cisplatin or Paclitaxel on the AGS wt and Resistant Cells

**Figure 11:**
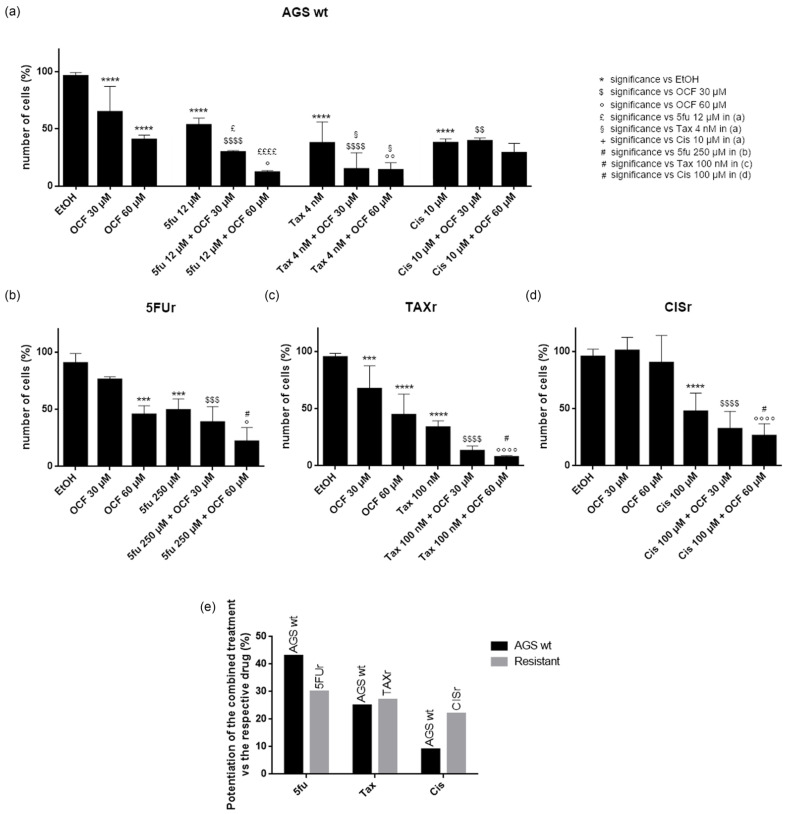
Effects of chemotherapeutic drugs in combination with the OCF on AGS wt and resistant cells. Cell count after 72 h treatment. AGS wt cells were treated with vehicle/OCF 30 µM/OCF 60 µM ± IC_50_ dose of 5-fluorouracil (12 µM)/Paclitaxel (4 nM)/cisplatin (10 µM) (**a**). 5FUr cells were treated with vehicle/OCF 30 µM/OCF 60 µM ± IC_50_ dose of 5-fluorouracil (250 µM) (**b**). TAXr cells were treated with vehicle/OCF 30 µM/OCF 60 µM ± IC_50_ dose of Paclitaxel (100 nM) (**c**). CISr cells were treated with vehicle/OCF 30 µM/OCF 60 µM ± IC_50_ dose of cisplatin (100 µM) (**d**). Representation of the percentage increase in efficacy between combined treatments (IC_50_ dose of drug and 60 µM OCF) *vs* the mono-treatment with the single IC_50_ dose of the drug in the different cell lines obtained from the previous experiments is shown in this figure (**e**). Data are presented as mean ± SD of three independent experiments. Meaning of the following significance symbols is specified on top-right of the figure: *** *p* < 0.001, **** *p* < 0.0001, $ *p* < 0.05, $$ *p* < 0.01, $$$ *p* < 0.001, $$$$ *p* < 0.0001, ° *p* < 0.05, °° *p* < 0.01, °°°° *p* < 0.0001, £ *p* < 0.05, ££££ *p* < 0.0001, § *p* < 0.05, # *p* < 0.05 (Two-way ANOVA Tukey’s multiple comparisons test).

In order to test a possible potentiation effect of the OCF in the toxicity of 5-fluorouracil, cisplatin or Paclitaxel on AGS wt and resistant cells, we decided to use drugs at the IC_50_ dose for each cell type, as determined in [37] and reported in Figure 11. We found that in the AGS wt cells, the 60 µM dose of the OCF potentiated the effect of all of the drugs, namely 5-fluorouracil, Paclitaxel and cisplatin, as assessed by the decrease in the viable cells (Figure 11a). The same potentiating activity was also found when the 60 µM dose of the OCF was combined with 5-fluorouracil or Paclitaxel in order to treat the 5FUr or TAXr cells, respectively (Figure 11b,c). The enhancing effect of the 60 µM dose of the OCF on the cisplatin-treated CISr cells (Figure 11d) is extremely interesting, since the 60 µM dose of the OCF alone was ineffective in the CISr. Therefore, the OCF is able to potentiate a drug toxicity in all AGS resistant cells (Figure 11e) and the latter discovery is important in view of the polychemotherapy approach used in GC.

## 4. Discussion

Several therapeutic approaches have been proposed in order to cure cancer, including chemotherapy and more recently, targeted therapy, both of which represent the most validated strategies. However, both regimens do not prevent important side effects in patients and possible relapses. Thus, it is urgent to verify whether natural compounds, with a low toxicity for the organism, but with evident anticancer properties, may be associated with standard chemotherapy.

In this study, we used an OC enriched EVO oil extract (OCF), in which the OC represents 55.1% of the total content. The OC concentration in the EVO oils is variable, ranging from 0.2 mg/kg to 498 mg/kg [41], depending on several factors, such as the cultivar and the production techniques [42], and it represents about 10% of the total phenolic compounds in the EVO oil [43,44]. The data regarding the OC bioavailability are very few, even if the OC metabolites have been found in human urine [45,46]. Low and chronic doses of OC are sufficient in order to induce its beneficial effects in the organism: in fact the daily intake of about 25–50 mL of EVO oil is equivalent to approximately 10 mg of OC, that corresponds to about 10% of a normal dose of ibuprofen [14]. The anticancer activity of OC concerns several aspects of cancer cell survival and progression. OC has been shown to induce a growth inhibition and cellular apoptosis targeting the extracellular signal-regulated kinases 1/2 (ERK1/2) and AKT signaling pathways in multiple myeloma cells [27]. By affecting the HGF/c-met pathway, OC caused the decrease of cell viability and malignancy in human breast cancer cells and in human prostate cancer cells [18,22,47]. While targeting the STAT3 pathway, OC reduced cell proliferation and progression of melanoma and of human hepatocellular carcinoma [24,25]. Further, the growth of different breast cancer cell lines was found to be inhibited by OC by reducing the mammalian target of the rapamycin (mTOR) [21].

In this study, we investigated the effect of the OCF on the gastric adenocarcinoma cell line AGS wt and the AGS drug resistant cells. Once it was established that the OCF showed a toxicity in normal cells starting from 60 µM (Figure 1), we used this concentration in order to evaluate the OCF sensitivity of AGS wt and AGS resistant cells. We found that the 60 µM dose of the OCF was effective in reducing the viability and cloning efficiency of AGS wt, 5FUr and TAXr cells, but not in CISr cells. The OCF treatment results in the cell cycle arrest, as confirmed by the p-Rb level decrease and the p21 expression increase. The p21 protein is a small protein that is responsible for the inhibition of the cell cycle and its main transcriptional regulator is p53 [48]. The p53 protein is a tumor suppressor and its activation can lead to different cellular responses including the cell cycle arrest, senescence and apoptosis. A p53 activation can occur after a cellular exposure to various stresses, such as DNA damage and the increase in the ROS determining the p21 up-regulation [49]. Plausibly, as also supported by the findings of *Cusimano* et al. [26], the increase in the p53 expression after the OCF treatment in the AGS cells correlates with the induction of the ROS levels, detected by the DCFDA staining in the AGS wt, 5FUr and TAXr cells (Figure 10a), but not in the CISr cells. The ROS are unstable molecules that contain oxygen and their overproduction correlates with DNA damage [50] and cellular apoptosis via a mechanism called oxidative stress [51]. Cisplatin is a platinum-based complex, known to form adducts with DNA, causing DNA strand breaks [52,53]; further, the same drug critically affects mitochondrial DNA, thereby inducing the overproduction of the ROS [54]. By studying the oxidant mechanism, we found that the AGS wt, 5FUr and TAXr cells showed lower levels of the antioxidant enzymes AKR1B1, AKR1B10, AKR1C1, AKR1C3 and GPX2 (Figure 10b), with respect to the CISr cells, which express very high levels of these enzymes. The upregulation of the antioxidant proteins found in the CISr, due to chronic exposure to cisplatin, can explain the CISr resistance to the production of the ROS induced by the OCF treatment.

The Aldo-keto reductase (AKR) family is a group of oxidoreductases that reduce carbonyl substrates, they are also involved in the metabolism of xenobiotic compounds. Mounting evidence supports the implication of the AKR in the acquisition of chemoresistance; especially, as summarized by *Matsunaga* et al. [55], AKR1B1, AKR1B10, AKR1C1, AKR1C2 and AKR1C3 were up-regulated in several tumors after their exposure to drugs. *Phoo* et al. [56] demonstrated that AKR1C1 and AKR1C3 were key molecules for the resistance to cisplatin in signet ring cells gastric carcinoma (KATO cells), by regulating their redox-dependent autophagy. In addition, *Chen* et al. [57] found an increase in AKR1C1, AKR1C2, AKR1C3 levels after a cisplatin administration for ovarian cancer, and *Ueda* et al. [58] documented the cisplatin-induced upregulation of AKR1C1, AKR1C2 in uterine cervical cancer. Likewise, the cisplatin resistance of ovarian, cervical and lung cancers has been reported to be mediated by AKR1C1 and AKR1C2 [59,60].

The anticancer properties of OC can be exploited in order to potentiate the toxicity of chemotherapeutic drugs, as reported by in vitro studies on breast cancer cells, with very satisfying results [61,62]. Here, we demonstrated that the OCF potentiated the IC_50_ dose of 5-fluorouracil, Paclitaxel and cisplatin of the AGS wt and 5FUr, TAXr and CISr drug-resistant AGS cells. On the contrary, the CISr cells were non-responsive to the OCF treatment alone, but when these cells were exposed to both cisplatin and the OCF, the combo-treatment succeeded in potentiating the drug efficacy, thereby suggesting that the use of the OCF may be useful for a complementary therapy (OCF + cisplatin) in cisplatin resistant cells.

In conclusion, the OCF showed very promising anticancer activity toward GC cells and, even more interestingly, it was also effective in resistant GC cells.

Therefore, we affirm that the OCF is a natural compound deserving of further study in order to increase the knowledge of the OC potential in order to improve cancer therapy, in vivo, in order to propose its use in clinical practice.

## Figures and Tables

**Table 1:** Composition of the OCF obtained by HPLC-DAD-MS.

Olea Extract Fraction (OCF)	
Compound	mg/g
hydroxytyrosol	12.38
tyrosol	8.29
elenolic acid	76.32
10-hydroxyoleocanthal	287.62
oleocanthal	189.16
oleuropein aglycone	95.44
ligstroside	23.29
secoiridoidic derivatives	171.99
**Total**	**864.50**

**Table 2:** List of primers.

Target Gene	Forward	Reverse
18S	5′-CGGCTACCACATCCAAGGAA-3′	5′-GCTGGAATTACCGCGGCT-3′
AKR1B1	5′-CCAACTTCAACCATCTCCAGGTG-3′	5′-GTCACCACGATGCCTTTGGACT-3′
AKR1B10	5′-CCAAGTCTGTGACACCAGCA-3′	5′-CGTTACAGGCCCTCCAGTTT-3′
AKR1C1	5′-TGCATAATGCCTGGGCTATCTT-3′	5′-AGGCCATGACAGTGTTTGAG-3′
AKR1C2	5′-GACCAGCCTTGGAAAGGTCA-3′	5′-AGACATGCAATCACGGAAGT-3′
AKR1C3	5′-ATGCCTGTCCTGGGATTTGG-3′	5′-GGCGGAACCCAGCTTCTATT-3′
GPX2	5′-CCCTTGCAACCAATTTGGAC-3′	5′-TCCTTCAGGTAGGCGAAGAC-3′

## Data Availability

Data is contained within the article.
